# The scale of repeat prescribing – time for an update

**DOI:** 10.1186/1472-6963-14-76

**Published:** 2014-02-19

**Authors:** Duncan R Petty, Arnold G Zermansky, David P Alldred

**Affiliations:** 1 School of Healthcare, University of Leeds, Leeds LS2 9UT, UK; 2 Bradford School of Pharmacy, School of Life Sciences, University of Bradford, Bradford BD7 1DP, UK

**Keywords:** Repeat prescribing, Prescribing analysis, Long-term medication

## Abstract

**Background:**

The NHS spends billions of pounds annually on repeat prescriptions in primary care, but data on their extent and use is out of date. Understanding the scale of repeat prescribing and for whom it is prescribed is important for the NHS to plan services and develop policies to improve patient care.

**Method:**

Anonymous data on prescription numbers and practice population demographics was obtained from GP computer systems in a large urban area.

Searches were conducted in November 2011 to identify the numbers of repeat items listed on individuals’ repeat lists by sex and age.

The proportion of all prescription items issued as repeats was identified by conducting searches on items issued as repeat and acute prescriptions.

**Results:**

In the year of study 4,453,225 items were issued of which 3,444,769 (77%) were repeats (mean 13 items per patient/annum) and 1,008,456 (23%) acute prescriptions (mean 3.9 items per patient per annum). The mean number of repeat Items per patient was 1.87 (range 0.45 ages 0-9 years; 7.1 ages 80-89 years). At least one repeat medicine was prescribed to 43% of the population (range 20% for ages 0-9; over 75% for ages 60+).

**Conclusion:**

A significant proportion of the population receive repeat prescriptions and the proportion increases with age. Whilst the proportion of repeat items to acute items has remained unchanged over the last two decades the number of repeat prescriptions items issued has doubled (from 5.8 to 13.3 items/patient/annum). This has implications for general practice workload, patient convenience, NHS costs and risk.

## Background

In the UK most NHS patients receive medicines intended for long-term use as "repeat prescriptions". These are prescription items that are generated without the need for a consultation from a list of authorised repeat medicines. It is a truth universally acknowledged [1,2] but not justified with any recent evidence, that the proportion of the population on repeat medication is high and increasing. As Avery notes [3], evidence on the prevalence of repeat medication is almost all out of date. Harris and Dajda used data from a four year period to 1994 to report that 48% of patients had at least one repeat medication and that repeats accounted for 75% of items and 81% of costs [4]. Since then there have been no data published. The recent King’s Fund report on the quality of GP prescribing had to use these 17-year-old data [2]. It is remarkable that there is such a dearth of data on an activity that exposes patients to the risks as well as the benefits of so many medicines and accounts for many billions of pounds of NHS resource.

This paper provides an update on the extent of repeat medication prescribing in a population of a large urban area in the North of England.

## Method

We employed a cross sectional design using data from general practices in Bradford and Airedale Primary Care Trust (PCT) in West Yorkshire. The PCT had 79 practices. We approached a convenience sample of 29 practices. These practices were used because they all had a practice pharmacy services enabling access to practice data within the time frame of the research. The population of the 29 practices were larger than non-study practices but did not differ in terms of age, sex and deprivation (see Table 1). Practice consent was obtained to use anonymous data on prescription numbers and practice population demographics. All practices in the PCT used SystmOne (TPP Ltd), which lists medicines authorised for repeat use on a "repeat template". We defined a repeat medicine as any medicine on a repeat template. Searches were conducted in November 2011 at practice population level to identify the numbers of repeat items listed on individual’s repeat template. The mean number of repeat prescriptions items per patient was calculated for each ten-year age/sex band by dividing the total number of repeat medicines listed by the population. To identify the total number of items issued per patient searches were constructed on SystmOne that identified whether an issue was an "acute medication" issue or issued from the patient’s "repeat template" in the financial year 2011/12. Repeat issues were then expressed as a proportion of all issues (repeats and acutes).

**Table 1:** Comparison of consenting (study) and other PCT practice demographics

	**Study practices**	**Other practices**
Number of practices	29	50
Total population	262,933	290,821
List size: mean (SD)	9066 (4359)	5816 (2837)
Age: mean (SD)	34 (5.0)	36 (5.8)
% female	50.1%	50.1%
Index of multiple deprivation: mean (SD)	34.4 (11.8)	37.7 (14.7)

## Results

### Patients, practices, total prescriptions issued

All 29 of the practices approached consented to using their data. A comparison of study practices’ verse non-study practices’ demographics in the same PCT is shown in Table 1.

The aggregate number of repeat prescription items listed on individual patients repeat lists was 483,431. In the year of study 4,453,225 items were issued of which 3,444,769 were repeat prescriptions (77%) (13.1 items per patient per annum) and 1,008,456 (23%) (mean 3.8 items per patient per annum) as acute prescriptions. The median percentage of repeat prescription items (of total prescription items) across all the age/sex bands was 77% (IQR 73-80%) (minimum 67%, maximum 87%).

### Patients on at least one medicine

**Figure 1:**
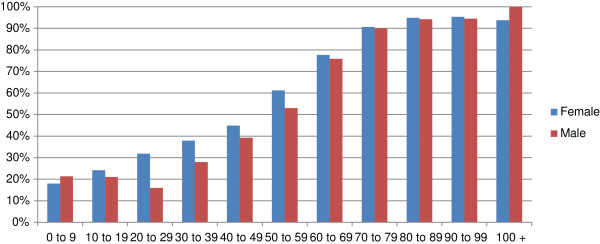
The percentage of patients receiving at least one repeat prescription, by age and sex.

Forty-three per cent of the population was prescribed at least one repeat medicine. This figure ranged from 20% for the 0-9 year olds to over 75% for the over 60s (Figure 1).

Overall (including patients on no medicines in the denominator) the mean number of repeats Items per patient on practice lists was 1.9.

### Number of repeat items by age and sex

**Figure 2:**
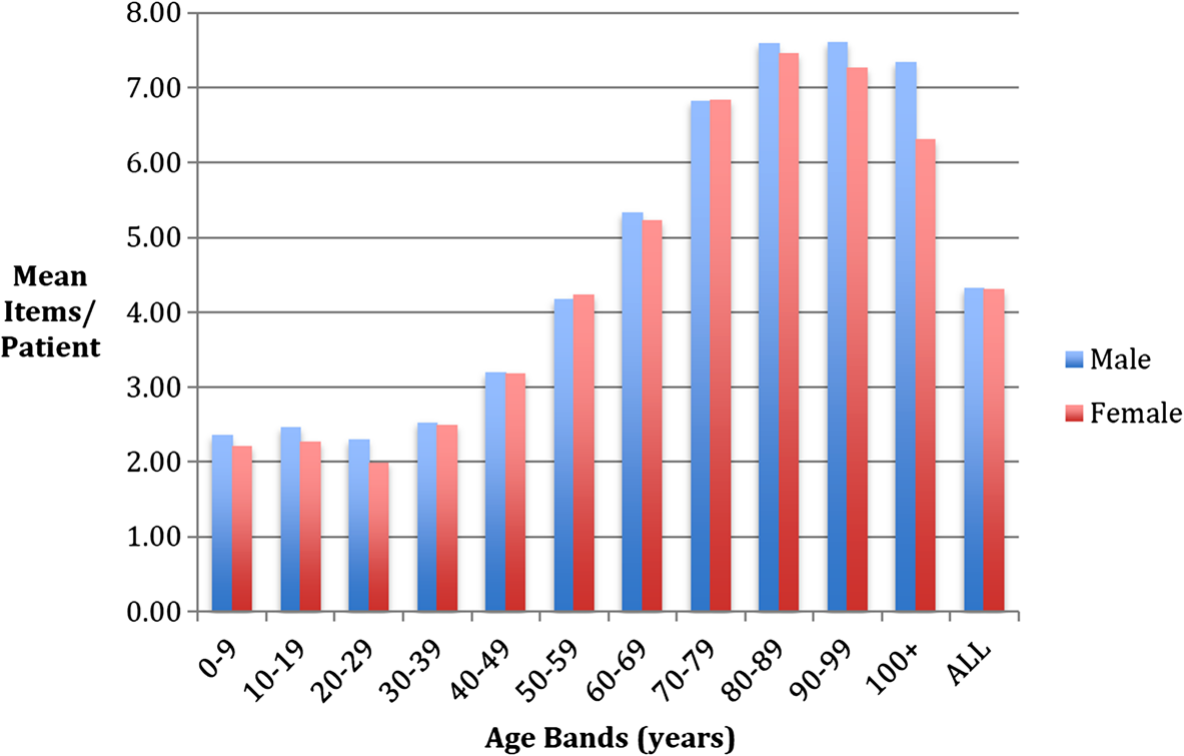
Mean number of medicines per patient on repeats.

Of those patients on at least one repeat medicine the mean number of repeat medicines per patient was 4.3 (Figure 2).

## Discussion

Our study is a comparatively small one compared with Harris and Dajda (who were able to sample data from the whole of England). Nonetheless we believe our sample, though only from one geographical area, is sufficiently large to allow guarded comparison, especially as there are no other currently available data. This study demonstrates the extent of change in both the total quantity of repeat prescribing items and the age sex distribution of their use since Harris and Dajda’s report of data from 17-18 years before (4). The proportion of patients on repeat medicines is lower than that reported by Harris and Dajda being 43% compared to 48%. The proportion of children and young adults on repeats in our study was 25% compared to 35% with Harris and Dajda’s study The proportion of older adults on repeats is the same ranging from around 75% in the 60+ group to over 90% in the over 70s. Close comparison between our study and that of Harris and Dajda is not warranted as our study used a local sample compared to a national sample.

It is only when the number of items per patient is compared that a large increase is seen. Our study recorded 13 issued items per patient per annum, compared with 5.8 issues for Harris and Dajda [4]. In England in 2011 an average of 18.7 prescription items were dispensed per head of population [5].

Many Primary Care Trusts in England have recommended restricting prescription length to 28 days on the advice of the National Prescribing Centre and the Department of Health [6] as a result prescription durations have been shown to have reduced between 1998 and 2009 [7]. The cost benefit of shorter prescription durations has been questioned [6,7] because of only a limited effect on reducing waste but with a large increase in dispensing costs [7]. The policy to reduce prescription length may also have resulted in more inconvenience for patients and a greater work load for them in ordering their supplies [8]. Our data shows that the majority of older people are on a repeat prescription and therefore are disproportionately affected by polices for 28 day prescribing.

The study was conducted in one large urban area in Northern England. Less than half of practices participated, possibly limiting external validity of data. Nonetheless, the number of practices and patients participating was sufficient to give a guide as to major prescribing trends. Although our study practices were larger than non-study practices they were similar in terms of age and deprivation scores, which are the two largest determinants of prescribing variation [9-11]. Although this study was on a relatively small scale in one English city we believe the prescribing rates are likely to be typical of the UK because our consenting practices covered a wide socio-economic and cultural range of population and included both small and large practices. A more complex study on a larger scale exploring the population and drug types prescribed in greater detail would be valuable to track the epidemiology of drug use.

Some practices do not record all long-term medication on repeat templates. In particular, oral contraception is often prescribed as a six month supply without adding it to the repeat template. Some practices are reluctant to include potentially harmful medicines that need frequent monitoring (such as methotrexate) on the repeat template.

Although we did not collect data about individual groups of drugs, the figures for overall dispensing of medicines from the NHS Business Authority give some indication of the growth areas [12]. Cardiovascular drugs and endocrine drugs are the areas of highest growth, and it is likely that the development of evidence-based guidelines, commencing with the National Service Framework for Coronary Heart Disease in 2000 and followed by NICE guidelines, SIGN guidelines and British Hypertension Society Guidelines to name only a few will have influenced the process. The Quality and Outcomes Framework for General Practice, which began in 2004, encouraged GPs to be more methodical and inclusive in case-finding and treatment, will also have fuelled change [13].

The implication of these evidence-based changes is that they represent quality prescribing. The more medicines taken, however, the greater the risk of harm from adverse drug events, and the reports of drug-related hospital admissions suggesting that over 6% of acute admissions result from therapeutic misadventure sound a warning of the need to be circumspect about polypharmacy [14,15]. The continuation of so many drugs into later ages also raises the question of whether they are still appropriate, particularly when evidence of efficacy or safety is seldom derived from clinical trials in the old and the potential to extend life expectancy may no longer be the priority. Therapeutic momentum, where drugs are continued beyond their need merely because no-one has had the thought (or perhaps the courage) to stop them is a potentially important risk.

All UK general practices have systems to electronically manage and generate repeat prescriptions. Previous studies have shown problems in the control of these systems including deficiencies in the authorisation and review of repeats by doctors [16] and the issuing of medicines (which are not repeats) by receptionists [1]. A report for the General Medical Council on the prevalence and causes of prescribing errors in general practice found a high frequency of errors [17]. One of their recommendations was that general practices review the procedures they have in place for repeat prescribing, medication monitoring and medication reviews. Since the majority of prescriptions are prescribed as repeats this recommendation would have a high impact of patient safety.

### Ethical approval

Ethical approval was obtained from the School of Healthcare Research Ethics Committee, University of Leeds.

## Conclusion

A significant proportion of the population receive repeat prescriptions and the proportion increases with age. Whilst the proportion of repeat items to acute items has remained unchanged over the last two decades the number of repeat prescriptions items issued has doubled (from 5.8 to 13.3 items/patient/annum). This has implications for general practice workload, patient convenience, NHS costs and risk.

## Competing interests

The authors declare that they have no competing interests.

## Authors’ contributions

DP had the idea for the study, designed the method for data collection, collected data and wrote the first draft of the paper. AZ contributed to the design of the study, checked some of the data, produced some of the tables and figures and contributed to the discussion. DA contributed to the design of the study and the writing of the manuscript. All authors read and approved the final manuscript.

## Pre-publication history

The pre-publication history for this paper can be accessed here:

http://www.biomedcentral.com/1472-6963/14/76/prepub
